# Supplementary material for: EFA6B regulates a stop signal for collective invasion in breast cancer
Source: Nat Commun. 2021 Apr 13;12:2198. doi: 10.1038/s41467-021-22522-4 (PMC8044243; doi:10.1038/s41467-021-22522-4)
Supplement: Supplementary file 1 — Supplementary Information [file 41467_2021_22522_MOESM1_ESM.pdf]

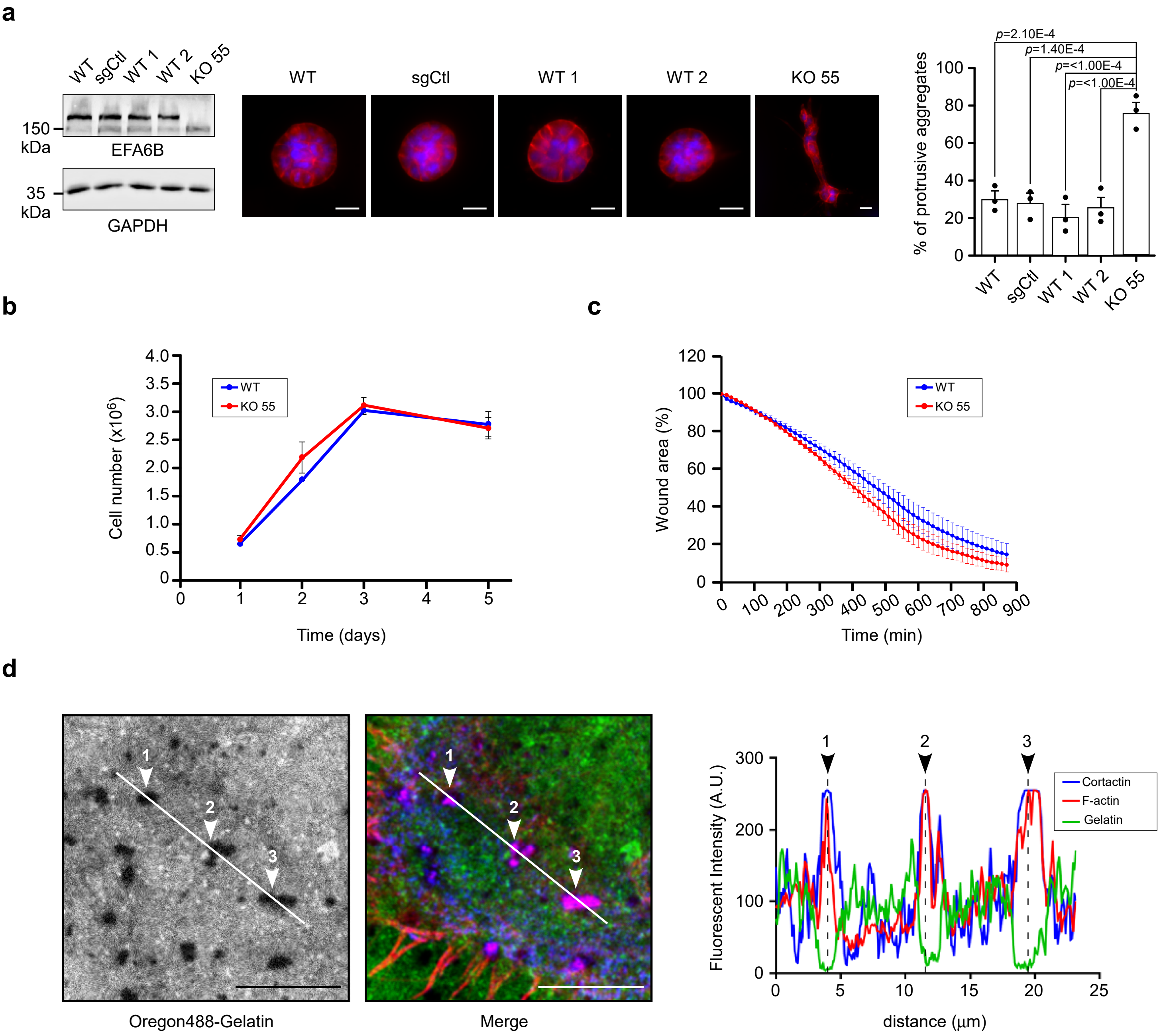

Supplementary Figure 1: **EFA6B knock-out does not affect cell proliferation nor migration but stimulates the formation of degradative invadopodia**. **a** Left panel: Expression of EFA6B in MCF10A WT, MCF10WT expressing the sgRNA control (sgCtl), two negative clones (WT1, WT2) and MCF10A KO55 cells analyzed by immunoblot. GAPDH served as a loading control. Middle panel: Representative images of the indicated cell lines placed in collagen for 4 days. The cell aggregates were processed for immunofluorescence to label the endogenous F-actin (red) and the nuclei (blue). Scale bars 20µm. Right panel: Quantification of the percentage of cell aggregates with invasive protrusions of the indicated MCF10A cell lines grown in collagen for 2 days. N=3, average ±SEM, one-way ANOVA test with Dunnett's multiple comparison p values. **b** MCF10A WT and EFA6B KO55 cells were plated at $5x10^5$ cells per well in a 12-well dish and counted over 5 days using the LUNA™ cell counter (Logos Biosystems). The graph represents the mean of 2 independent experiments ±SD. **c** MCF10A WT and EFA6B KO55 cells were plated at confluency in a 12-well dish (106 cells per well). The next day a wound was performed using a pipet tip. After a wash with complete medium, the closure of the wound was followed overtime by phase contrast video-microscopy using a Cytation5 (Biotek). The images taken every 15min were processed and the area of the wound closure calculated using the software ImageJ. The graph represents the percentage of closure relative to the surface area of the wound at t=0 of 5 independent experiments performed in triplicates ±SEM. **d** EFA6B KO55 cells were stained for F-actin (red) and cortactin (blue) 48h after plating on Oregon488-gelatin (green) coated coverslips. The left image is the Oregon488-gelatin staining to visualize the digested spots. The right image is the merge of all three markers to visualize the co-localization of cortactin with F-actin, appearing in purple, coincidental with the spots of digested gelatin. Scale bars 10µm. The graph shows the fluorescent intensity of all three markers over the indicated line scan on the image. Arrowheads point to three invadopodia selected along the line scan. Source data are provided as a Source Data file.

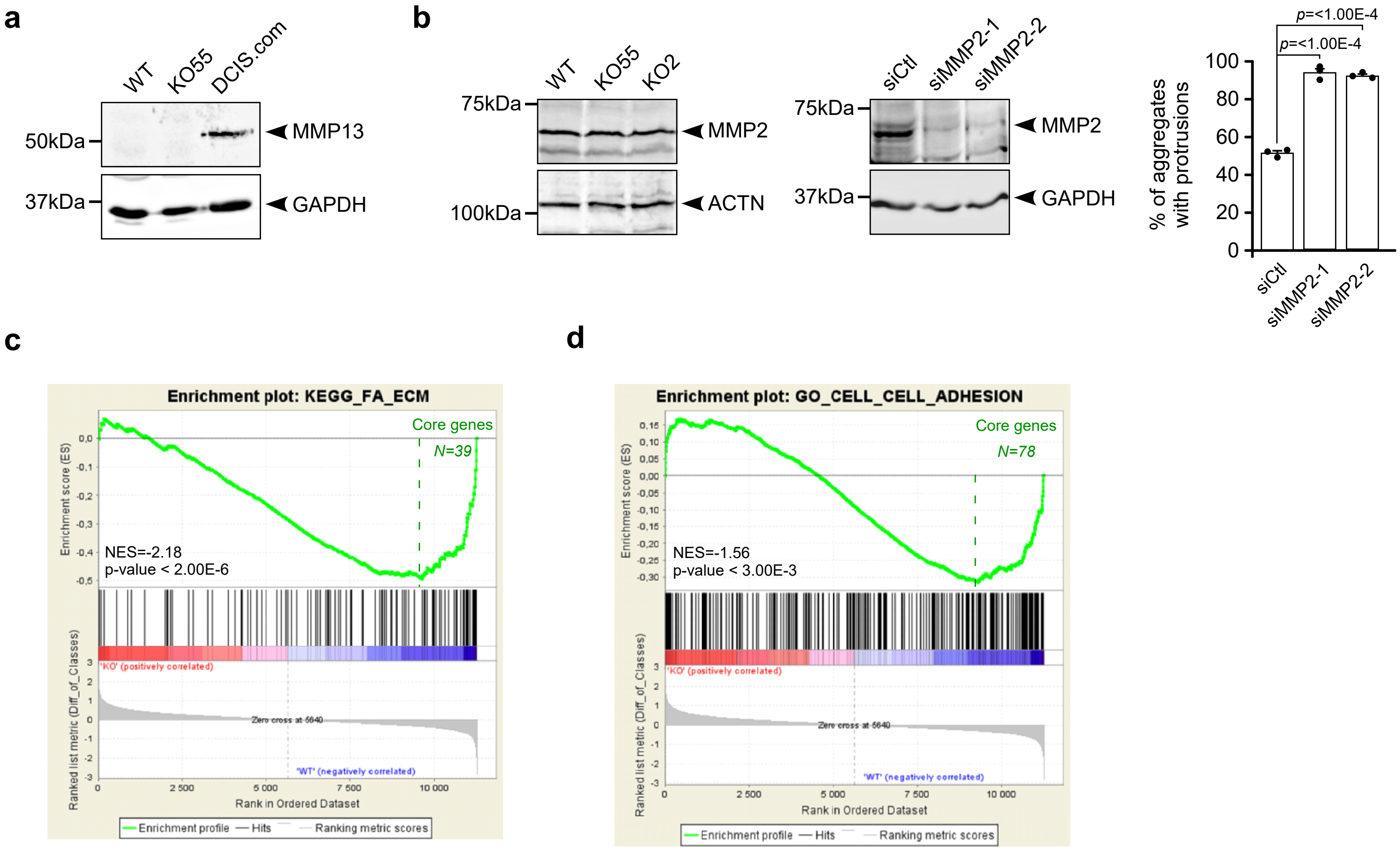

Supplementary Figure 2: **a** MMP13 is not expressed in MCF10A cells. Expression of MMP13 by immunoblot in MCF10A WT, EFA6B KO55 and the control positive cell line MCF10ADCIS.com. MMP13 antibody was from R&D systems (MAB511). GAPDH served as a loading control. **b** MMP2 depletion accelerates invasion. Left panel: Expression of MMP2 analyzed by immunoblot in MCF10A WT, EFA6B KO55 and KO2 cells. Actinin (ACTN) served as a loading control. Middle panel: Expression of MMP2 analyzed by immunoblot two days post-transfection of EFA6B KO55 cells with the indicated siRNAs (Dharmacon ON-TARGETplus, siRNA-1: ACAAGAACCAGAUCACAUA, siRNA-2: GGAAUGCCAUCCCCGAUAA). MMP2 antibody was from Sigma (HPA001939). GAPDH served as a loading control. Right panel: quantification of the percentage of aggregates (n=100) with invasive protrusions grown in 3D-collagen (3mg/ml) for 36 hrs after transfection with the indicated siRNAs. N=3, average ±SEM, one-way ANOVA test with Dunnett's multiple comparison p values.
Gene set enrichment analysis (GSEA) of *PSD4* KO versus WT MCF10A cells, showing enrichment in the WT cells of the "Matrisome+receptors" gene set (KEGG_FA_ECM, including genes of the KEGG gene sets Focal Adhesion ('FA'), ECM-receptor interaction ('ECM') and the human matrisome database) (**c**), and of the Gene Ontology "cell-cell adhesion" signature (**d**). GSEA p-values were based on permutation-generated null distribution of enrichment scores (ES) within the Broad Institute software, naturally two-sided test. Benjamini-Hodgberg method was used to correct for multiple comparisons.Source data are provided as a Source Data file.

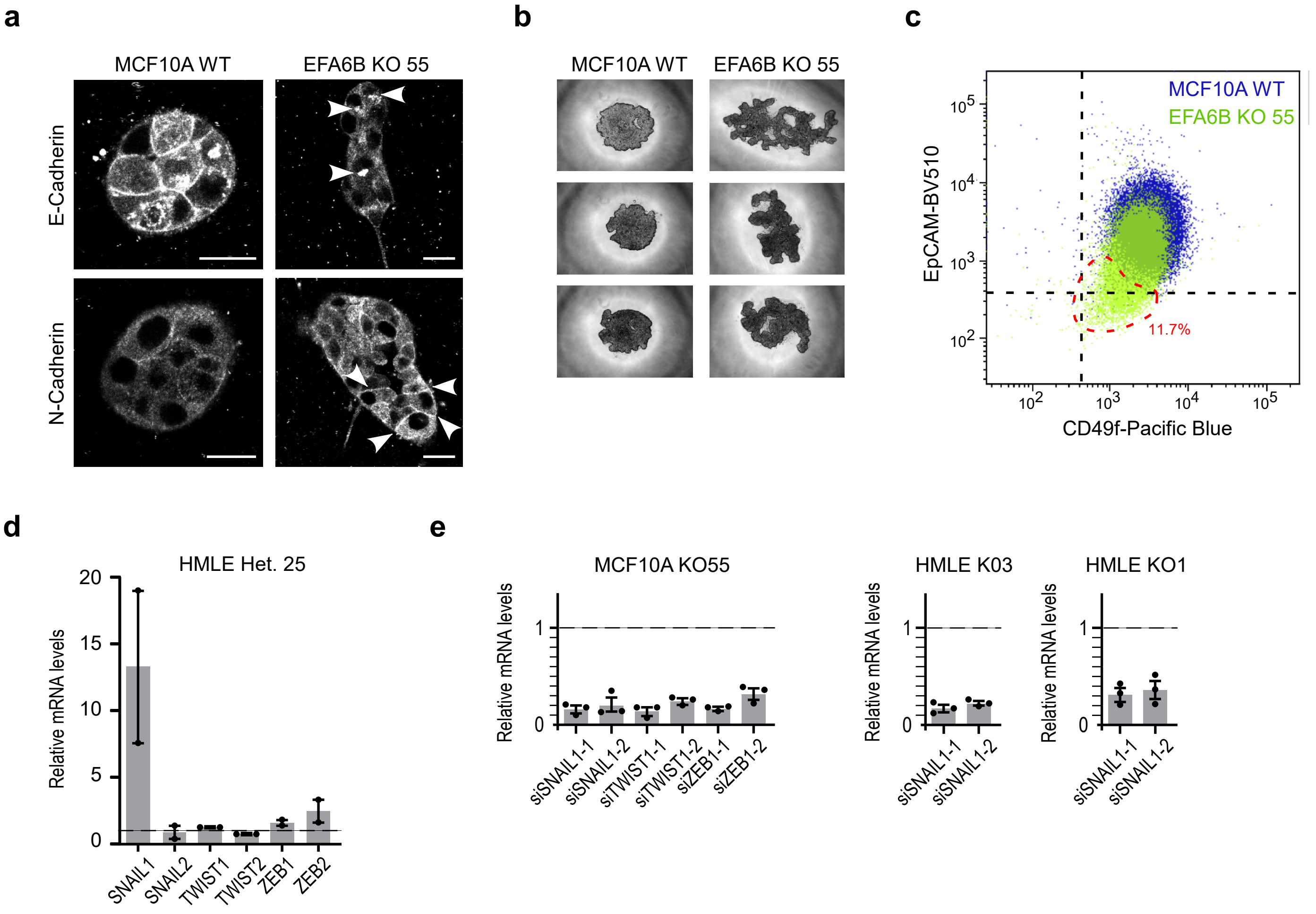

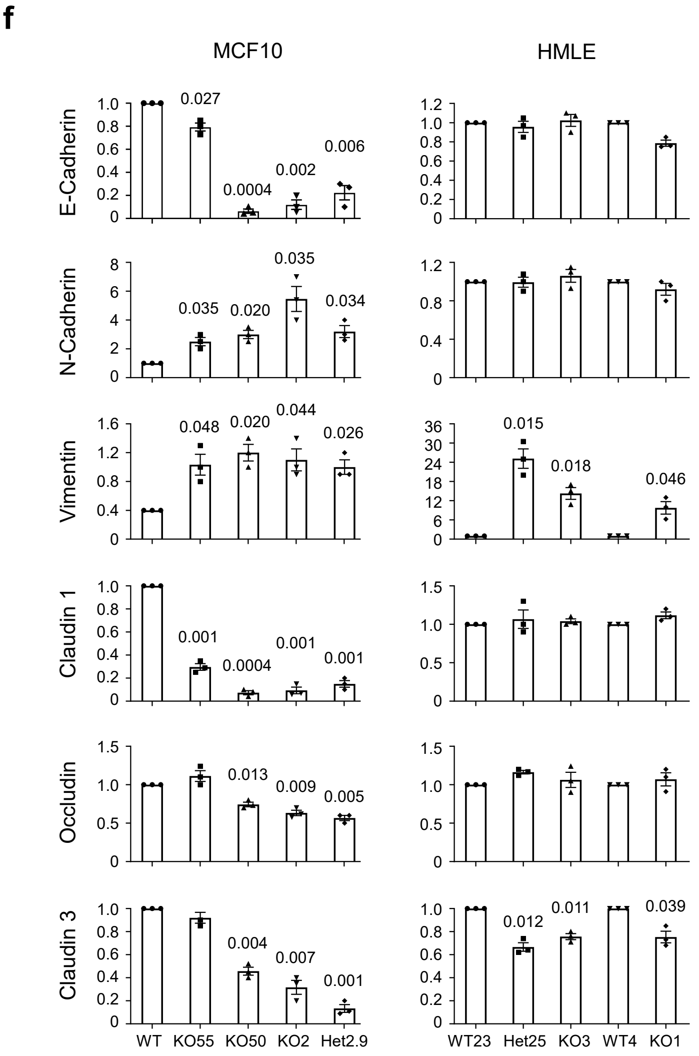

Supplementary Figure 3: EFA6B knock-out induces EMT. **a** Representative images of the indicated spheroids grown 2 days in collagen I stained for E-cadherin or N-cadherin. Arrowheads in upper right panel indicate intracellular E-Cadherin. Arrowheads in lower right panel indicate plasma membrane localized N-cadherin. Scale bars 20µm. **b** Representative bright field phase contrast images of the indicated cells grown 2 days in hanging drops, N=6. **c** The MCF10A WT (blue) and EFA6B KO55 (green) cells were labeled for the cell surface markers EpCAM and CD49f. The red dotted line circles a new de-differentiated population EpCAM$^{-/low}$ and CD49f^{low} (11.7%) appearing in the EFA6B KO55 cell line. **d** Expression of EMT-associated genes by RTqPCR analysis in HMLE Het.25 cells normalized to HMLE WT23, N=2, average ± range. **e** Expression of the indicated EMT-TF genes by RTqPCR analysis 2 days post-transfection in MCF10A EFA6B KO55, HMLE EFA6B KO3 and KO1 cells with the indicated corresponding specific siRNAs normalized to siCtl. N=3, average ± SEM **f** Quantification of the immunoblot presented in Figure 5a,c. Intensities of signal normalized to the corresponding WT sample. N=3, average ±SEM, two-tailed Student's t-test *p* values, non-significant when not indicated. Source data are provided as a Source Data file.

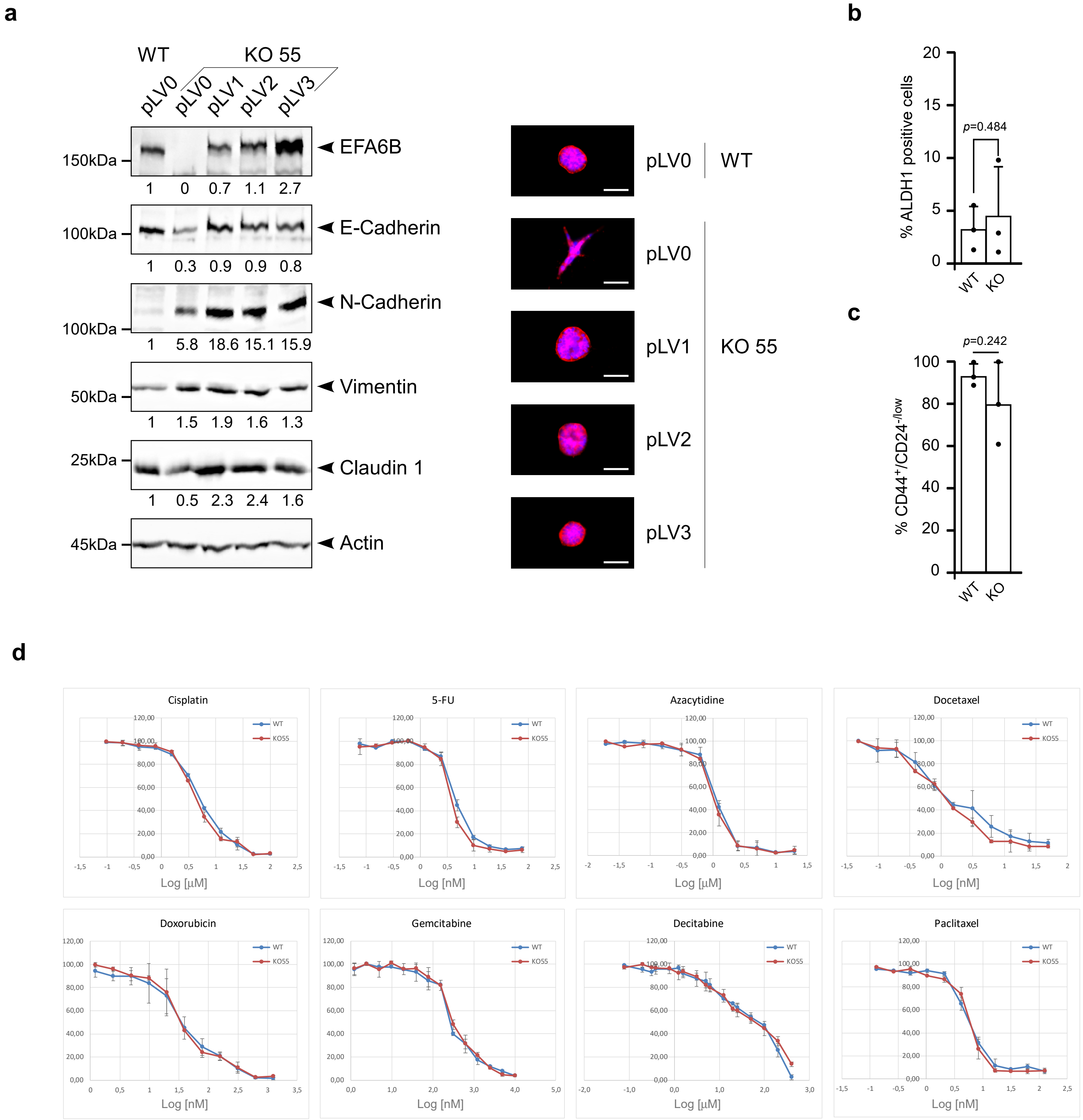

Supplementary Figure 4: EFA6B knock-out induces a partially reversible EMT without the acquisition of stemness characteristics. **a** Partial EMT reversal upon expression of exogenous EFA6B by lentiviral infection. The empty lentiviral plasmid pLV-Neo-CMV (pLV0) was used as a negative control, and expressed in MCF10A WT or EFA6B KO55 cells. pLV-EFA6B at MOI 1/50 (pLV1), 1/25 (pLV2) or 1/10 (pLV3) was used to express increasing amounts of EFA6B in KO55 cells. Left panel: expression of the indicated proteins analyzed by immunoblot. Actin served as a loading control. N=3. Right panel: representative images of the corresponding cell aggregates placed in collagen for 4 days. The cells were processed for immunofluorescence to label the endogenous F-actin (red) and the nuclei (blue). Scale bars 20µm. **b** Percentage of ALDH1 positive cells in MCF10A WT and EFA6B KO55 cell lines analyzed by FACS. N=3; average ±SD, two-tailed Student's t-test *p* value. **c** Percentage of $CD44^{+}/CD24^{-/low}$ cells in MCF10A WT and EFA6B KO55 cell lines analyzed by FACS. N=3; average ±SEM, two-tailed Student's t-test *p* value. **d** The cell viability of both MCF10A WT and EFA6B KO55 cells after three days of drug exposure at the indicated concentrations was determined by alamar blue-based assay (Invitrogen). Fluorescence intensity was measured at 530nm and 590nm wavelength for excitation and emission using a microplate reader (Infinite® M1000 Pro, TECAN). The graph curves represent the mean percentage of viability relative to the respective untreated controls of three independent experiments ±SEM. Source data are provided as a Source Data file.

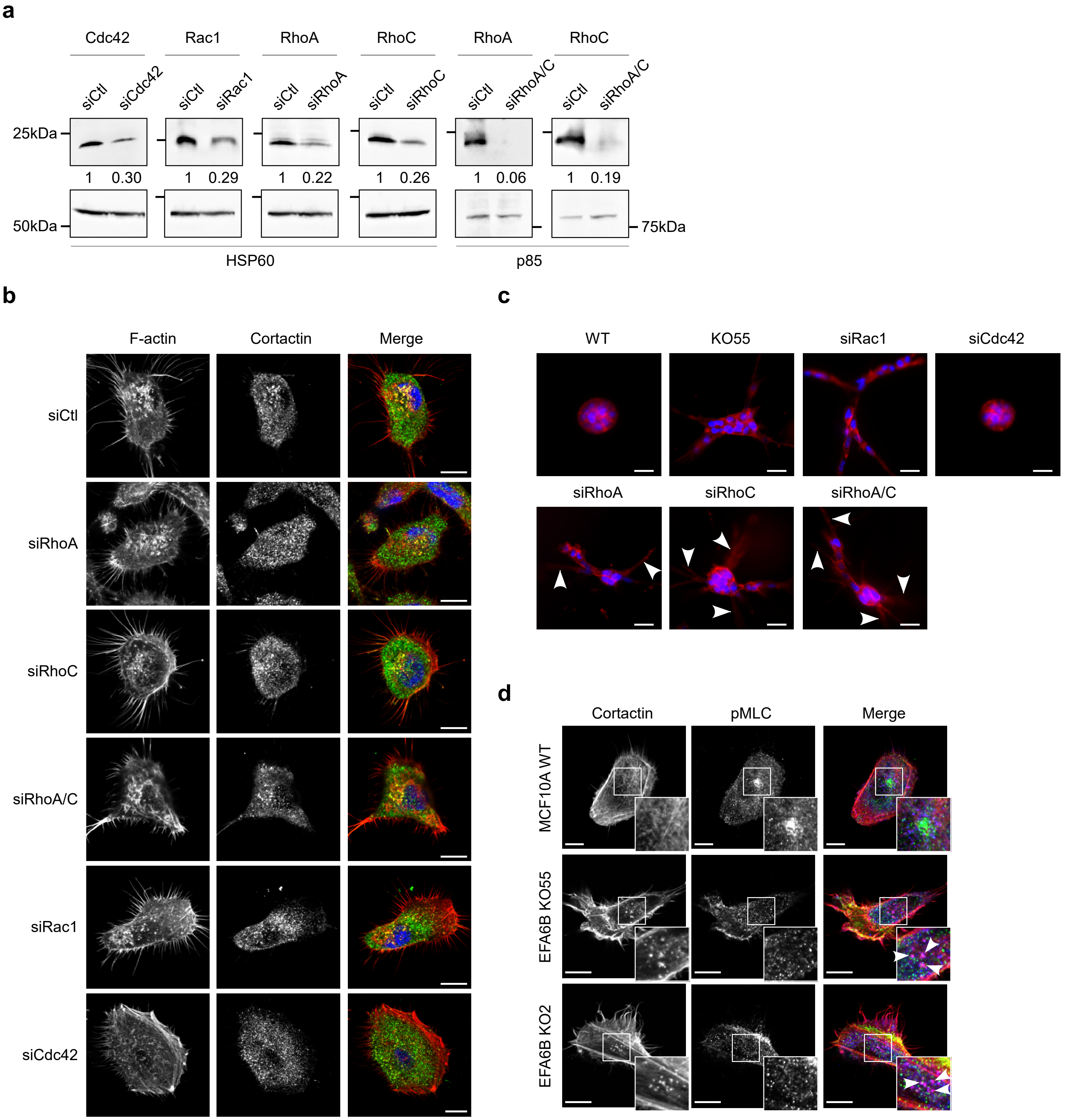

Supplementary Figure 5: Depletion of RHOA, RHOC, RAC1 or CDC42 all altered cell morphology but only depletion of CDC42 inhibited invasion in collagen. **a** Expression of the indicated proteins analyzed by immunoblot two days post-transfection of EFA6B KO55 cells with the indicated siRNAs. HSP60 or p85 served as a loading control. **b** Representative images of EFA6BKO55 cells transfected with the indicated siRNAs and stained for cortactin (green), F-actin (red) and nuclei (blue). The right image is the merge of all three markers. The co-localization of cortactin with F-actin appearing in orange indicates the presence of invadopodia. Scale bar 10µm. **c** Representative images of the corresponding cells shown in b) placed in collagen for 4 days. The cell aggregates were processed for immunofluorescence to label the endogenous F-actin (red) and the nuclei (blue). Scale bars 20µm. Arrowheads point to long membrane protrusions. **d** pMLC is not seen enriched in invadopodia. Representative images of the indicated cells grown 2 days on collagen I-coated coverslips stained for cortactin (red), pMLC (green) and F-actin (blue). The third panel is a merge of all three markers. The purple color shows the co-localization of cortactin (red) and F-actin (blue) in invadopodia, some of which indicated by arrowheads. The large inset is a 2X zoom-in image of the indicated area. Scale bars 10µm. Source data are provided as a Source Data file.

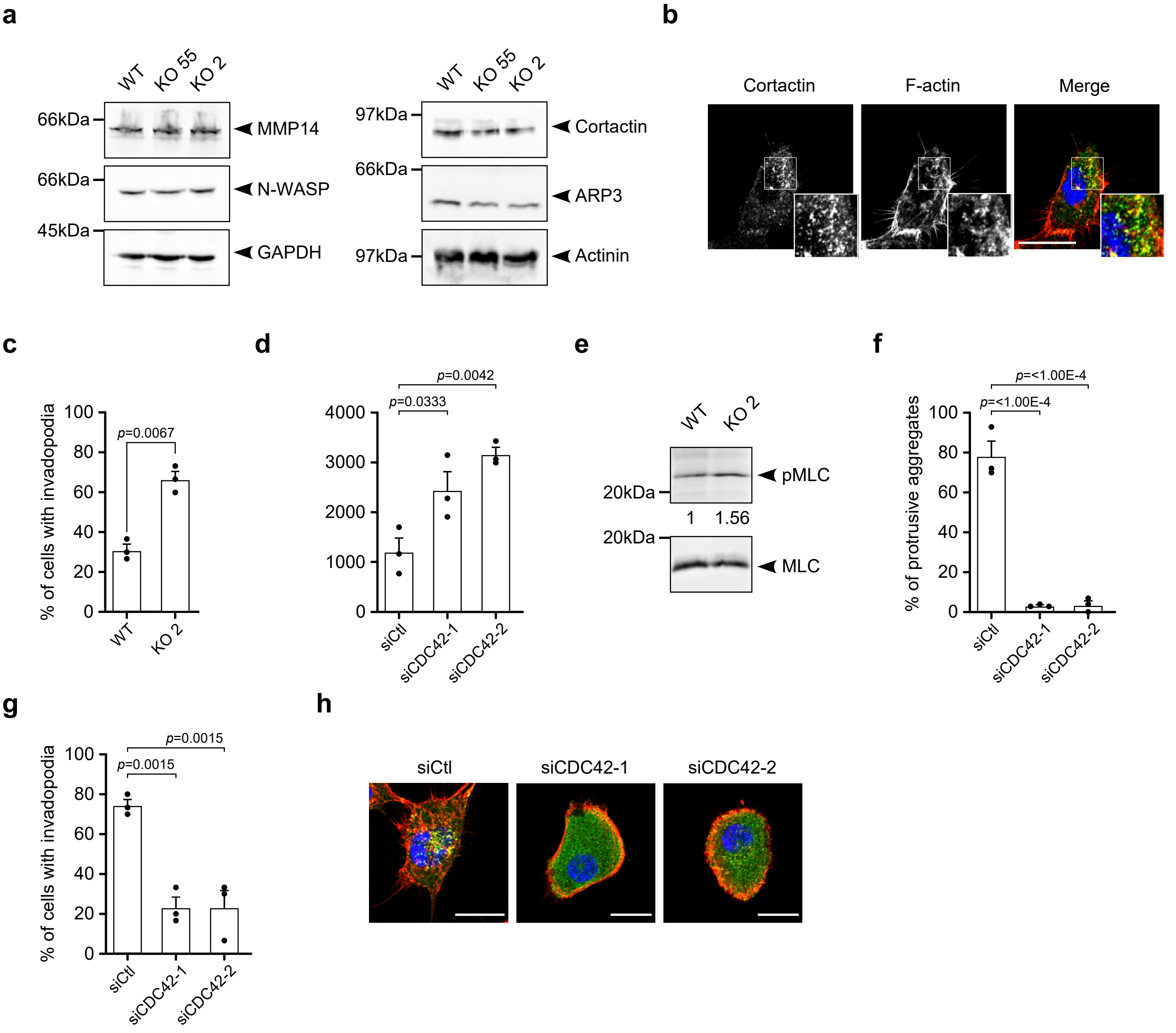

Supplementary Figure 6: EFA6B KO2 cells display contractility and invasive properties similar to the KO55 cells. **a** Expression of the indicated proteins analyzed by immunoblot in MCF10A WT, EFA6B KO55 and EFA6B KO2 cells. **b** Representative images of the EFA6B KO2 cells grown 2 days on collagen I-coated coverslips and stained for cortactin (green), F-actin (red) and nuclei (blue). The large inset is a 2X zoom-in image of the indicated area. Scale bar 20µm. **c** Quantification of the percentage of indicated cells (n=30) displaying invadopodia. N=3, average ±SEM, two-tailed Student's t-test *p* value. **d** Quantification of the contractility of EFA6B KO2 cells transfected with the indicated siRNA evaluated by a collagen gel contraction assay. Values are mean surface area of the collagen gel ±SEM. N=3, one-way ANOVA test with Dunnett's multiple comparison p values. **e** Expression of pMLC and total MLC analyzed by immunoblot in MCF10A WT and EFA6B KO2 cells, N=2. **f** Quantification of the percentage of aggregates with invasive protrusions of EFA6B KO2 cells grown in collagen I for 2 days post-transfection with the indicated siRNAs. N=3, average ±SEM, one-way ANOVA test with Dunnett's multiple comparison p values. **g** Quantification of the percentage of invadopodia in EFA6B KO2 cells grown in collagen I for 2 days post-transfection with CDC42 targeted siRNAs . N=3, average ±SEM, one-way ANOVA test with Dunnett's multiple comparison p values. **h** Representative images of EFA6B KO 2 cells transfected with the indicated siRNAs and stained for cortactin (green), F-actin (red) and nuclei (blue). Scale bars 20µm. Source data are provided as a Source Data file.

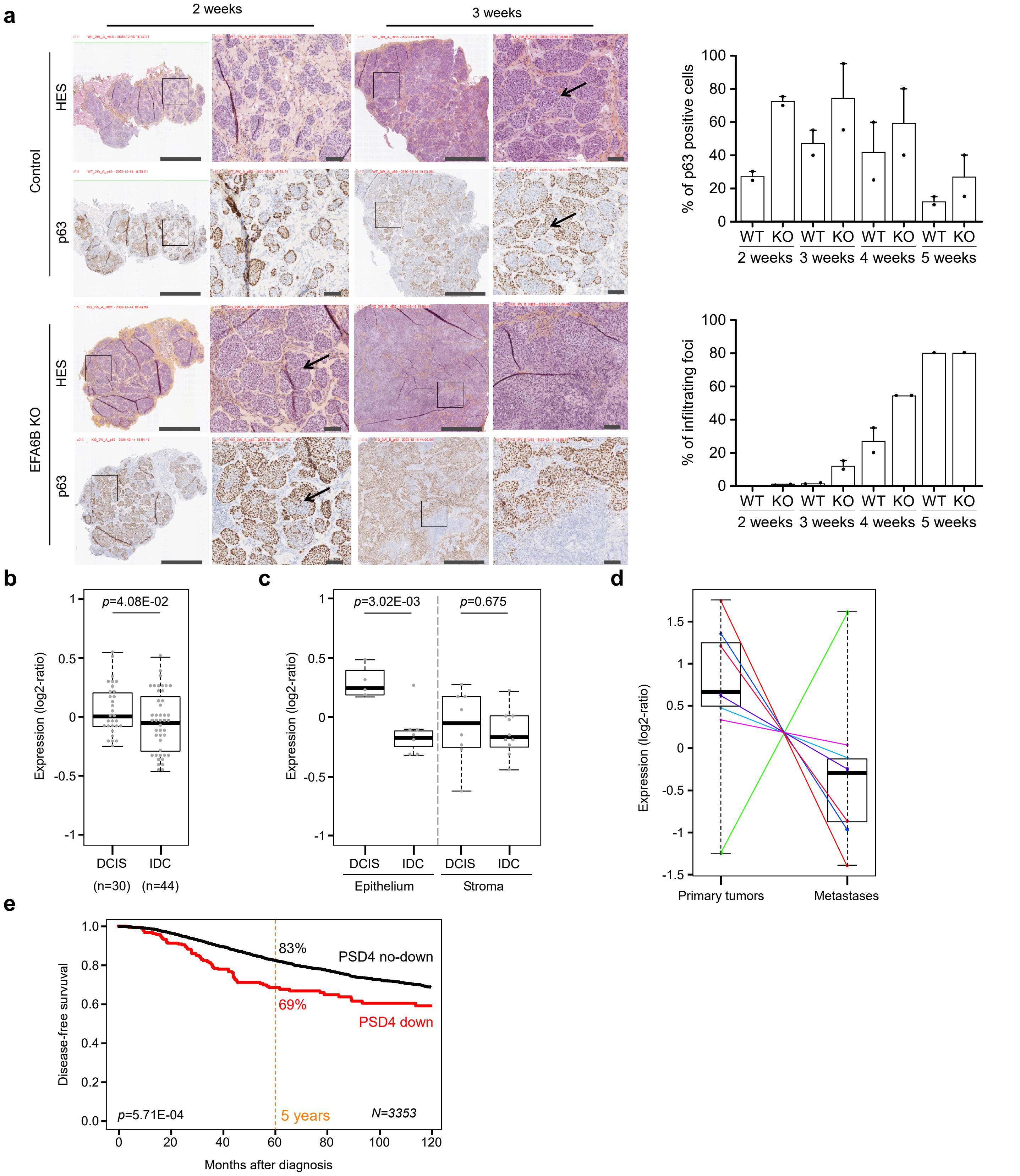

Supplementary Figure 7: EFA6B KO in MCF10DCIS.com cells stimulates tumor infiltration, and EFA6B/*PSD4* expression is down-regulated in human clinical TNBC samples endowed with invasive properties. **a** Representative images of control MCF10DCIS.com and EFA6B KO xenografts sequential sections analyzed by hematoxylin-eosin-safran staining and immunohistochemistry for the expression of p63. Arrows point to invasive tumors visible at week 2 or week 3 post-injection of EFA6B KO or control cells, respectively. Scale bars 1mm. The right panels are zoom-in image of the indicated area, scale bars 100μm. Right: Quantification of the percentage of p63^{+}cells (top panel) and infiltrating foci (bottom panel) from two sections of two different mice, except at 5 weeks where infiltrating foci were quantified on one section. **b**, **c** and **d** : for each box and whisker plot, the median value (50th percentile) and interquartile ranges (25th and 75th percentile) are indicated; points outside the whisker boundaries are defined as outliers. **b** Expression of *PSD4* in DCIS compared to IDC. The *p*-value is for the Two-tailed Student's t-test. **c** Expression of *PSD4* is selectively decreased in the epithelial compartment of DCIS (epithelium DCIS n=4, IDC=7; stroma DCIS n=8, IDC n=10). The *p*-values are for the two-tailed Student's t-test. **d** Expression of *PSD4* in primary tumors compared to paired metastases (n=9 pairs). The *p*-value is for the paired Mann-Whitney test. **e** Kaplan-Meier curves for DFS according to *PSD4* expression. The *p*-value is for the log-rank test. Source data are provided as a Source Data file.

Supplementary Figure 8: Flow cytometry gating strategy

Figure 2a

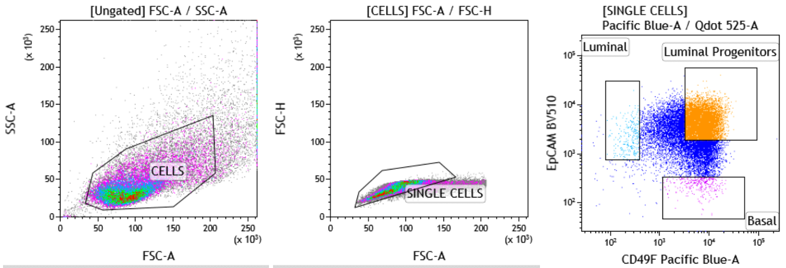

Gate	Number	%Gated
All	26,286	100.00
CELLS	22,280	84.76

Gate	Number	%Gated
All	22,280	100.00
SINGLE CELLS	17,077	76.65

Gate	Number	%Gated
All	17,077	100.00
Basal	292	1.71
Luminal	246	1.44
Luminal Progenitors	8,328	48.77

Figure 4a

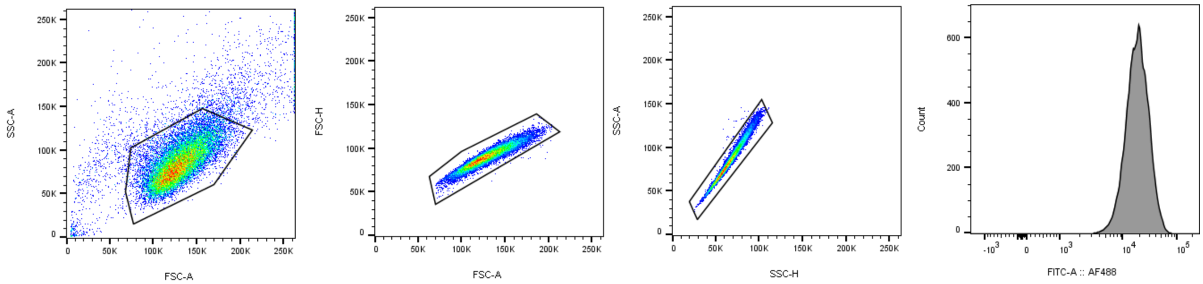

MCF10 WT_B4 MOUSE.fcs
Ungated
22175
100%

MCF10 WT_B4 MOUSE.fcs
MCF10A cells
19898
89.7%

MCF10 WT_B4 MOUSE.fcs
Single Cells
19878
99.9%

MCF10 WT_B4 MOUSE.fcs
Single Cells 2
19852
99.9%

Supp. Fig.3c

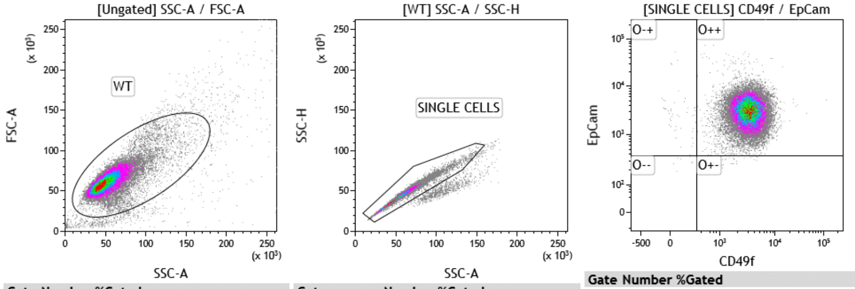

Gate	Number	%Gated
All	32,023	100.00
WT	31,153	97.28

Gate	Number	%Gated
All	31,153	100.00
SINGLE CELLS	30,306	97.28

Gate	Number	%Gated
All	30,306	100.00
O--	7	0.02
O-+	54	0.18
O+-	33	0.11
O++	30,212	99.69

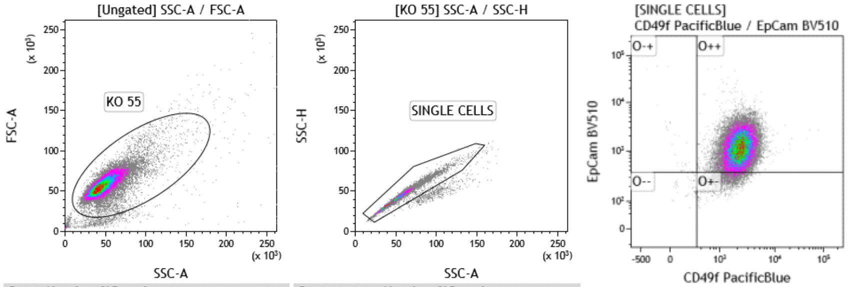

Gate	Number	%Gated
All	31,837	100.00
KO 55	30,944	97.20

Gate	Number	%Gated
All	30,944	100.00
SINGLE CELLS	30,428	98.33

Gate	Number	%Gated
All	30,428	100.00
O--	44	0.14
O-+	34	0.11
O+-	1,812	5.96
O++	28,538	93.79

Supplementary Table 1: List of four BC data sets included in the study

Reference	Source of data	N° of samples	Technological platform	N° of probe sets	N° of samples used
Jonsson et al., BCR 2010	GEO: GSE22133	359	Swegene H_v2.1.1 55K	55K	346
TCGA, Nature 2012	TCGA Data Portal - BRCA -	1215	Illumina, RNAseq V2	20K	1092
Ellis et al., Nature 2012	GEO: GSE29442, GSE35186	201	Agilent-014850 4x44K	44K	201
Curtis et al., Nature 2012	METBARIC: EGA: EGAS00000000083	2136	Illumina HT 12	49K	1974
	TOTAL	3911			3613

Supplementary Table 2: Correlations of PSD4 expression with clinico-pathological features of BC

Characteristics	N	PSD4 expression down (N=306)	PSD4 expression no down (N=3307)	p-value
Age at diagnosis, years				**6.68E-05**
≤50	753	93 (33%)	660 (22%)	
>50	2514	191 (67%)	2323 (78%)	
Pathological grade				**2.86E-11**
1	232	7 (4%)	225 (10%)	
2	1007	35 (22%)	972 (45%)	
3	1087	116 (73%)	971 (45%)	
Pathological lymph node status (pN)				0.752
negative	1522	134 (51%)	1388 (50%)	
positive	1509	127 (49%)	1382 (50%)	
Pathological tumor size (pT)				**3.86E-04**
pT1	1189	75 (27%)	1114 (38%)	
pT2	1699	169 (61%)	1530 (53%)	
pT3	292	35 (13%)	257 (9%)	
Pathological type				**3.18E-03**
ductal	2505	230 (81%)	2275 (76%)	
lobular	383	16 (6%)	367 (12%)	
other	377	38 (13%)	339 (11%)	
Molecular sutbype, mRNA*				**6.36E-50**
HR+/HER2-	2569	112 (37%)	2457 (75%)	
HER2+	398	49 (16%)	349 (11%)	
TN	628	142 (47%)	486 (15%)	
5-year DFS [95% CI]	3353	69% [62-76]	83% [81-84]	**5.71E-04**
Follow-up median, months (min-max)	3353	38 (1-232)	73 (1-382)	**2.12E-08**

*, status defined as previously described (Zangari et al,Cancer Res 2014)

p-value were based on Student t-test for continuous variables, Fisher's exact test for categorical variablesand Log-rank test for the DFS. All statistical tests were two-sided at the 5% level of significance.

Supplementary Table 3: List of primary antibodies used in this study

Antigen	Antibody source and reference	Application (dilution from stock or concentration)
Integrin-β1	Santa Cruz, sc-53711	Immunoblot 1/200
Integrin-β1	Santa Cruz, sc-13590	Flow cytometry 1/200<br>Immunofluorescence 1/100<br>Blocking antibody 7μg/ml
Integrin-β4	BD Biosciences, 611232	Immunoblot 1/200<br>Immunofluorescence 1/100
Integrin-β4	BD Biosciences, 555719	Flow cytometry 1/200
Integrin-β4	Millipore, MAB2059	Blocking antibody 30μg/ml
Integrin-α2	Santa Cruz, sc-53502	Flow cytometry 1/200<br>Immunofluorescence 1/100
Integrin-α2	Millipore, MAB1950	Blocking antibody 14μg/ml
Integrin-α3	Millipore, MAB2057	Flow cytometry 1/200<br>Immunofluorescence 1/100<br>Blocking antibody 30μg/ml
Integrin-α6	BD Biosciences, 555734	Flow cytometry 1/200<br>Immunofluorescence 1/100<br>Blocking antibody 30μg/ml
EpCAM	BD Biosciences, 563181	Flow cytometry 1/250
CD49f	BD Biosciences, 562582	Flow cytometry 1/1000
CD24	BD Biosciences, 561644	Flow cytometry 1/250
CD44	BD Biosciences, 560568	Flow cytometry 1/125
N-cadherin	BD Biosciences, 610921	Immunofluorescence 1/50<br>Immunoblot 1/500
E-cadherin	Thermo Scientific, 33-4000	Immunofluorescence 1/50
E-cadherin	BD Biosciences, 610182	Immunoblot 1/200
Vimentin	Sigma, V6389	Immunoblot 1/100
Claudin 1	ZYMED Laboratories, 51-9000	Immunoblot 1/100
Claudin 3	Millipore, 2819163	Immunoblot 1/200
Occludin	Thermo Scientific, 40-4700	Immunoblot 1/100
ARF1	Novus Biologicals, NB100-55421	Immunoblot 1/200
ARF5	Abnova, H00000381-M01	Immunoblot 1/200
ARF6	Gift from Dr. Bourgoin	Immunoblot 1μg/ml
EFA6A	Gift from Dr. Sakagami	Immunoblot 1μg/ml
EFA6B	Sigma, HPA034722	Immunoblot 1/200
EFA6D	Gift from Dr. Sakagami	Immunoblot 1μg/ml
MMP-14	Millipore, MAB3328	Immunoblot 1/100
Cortactin	Millipore, 05-180	Immunofluorescence 1/100<br>Immunoblot 1/200
N-WASP	Cell Signaling, 4848	Immunoblot 1/200
ARP3	BD Biosciences, 612234	Immunoblot 1/200
pMLC	Cell Signaling, 3671 & 3675	Immunoblot 1/50<br>Immunofluorescence 1/25
MLC	Sigma, M4401	Immunoblot 1/100
CDC42	BD Biosciences, 610928	Immunoblot 1/200
RAC1	BD Biosciences, 610650	Immunoblot 1/200
RHOA	Santa Cruz, sc-418	Immunoblot 1/200
RHOC	Cell Signaling, 3430	Immunoblot 1/200
ROCK 1	Santa Cruz, sc-17794	Immunoblot 1/50
ROCK 2	Santa Cruz, sc-398519	Immunoblot 1/200
P63	Diagomics, BSB3606	Immunohistochemistry 1/50
Actin	Sigma, A4700	Immunoblot 1/500
Actinin	Sigma, A5044	Immunoblot 1/500
GST	GE Healthcare, 27-4577-01	Immunoblot 1/1000
Hsp60	Sigma, SAB4501464	Immunoblot 1/200
p85	Millipore, ABS1856	Immunoblot 1/200
GAPDH	Sigma, G9545	Immunoblot 1/500

Supplementary Table 4: List of RT-qPCR oligonucleotides used in this study

Oligo name	*Sequence forward (5'-3')*	*Sequence reverse (5'-3')*
**E-Cadherin**	TGCCCAGAAAATGAAAAAGG	GTGTATGTGGCAATGCGTTC
**N-Cadherin**	ACAGTGGCCACCTACAAAGG	CCGAGATGGGGTTGATAATG
**Vimentin**	GAGAACTTTGCCGTTGAAGC	GCTTCCTGTAGGTGGCAATC
**CK14**	GGAAGCCGACATCAATGG	GCCTCTCAGGGCATTCATC
**ITGA6**	GATGCCACATATCACAAGGC	TGCATCAGAAGTAAGCCTCTCT
**Occludin**	TCAGGGAATATCCACCTATCACTTCAG	CATCAGCAGCAGCCATGTACTCTTCAC
**Claudin 7**	AGAGCACGGGGATGATGAG	CACCCATGGCTATACGGGC
**Snail 1**	GCTGCAGGACTCTAATCCAGA	ATCTCCGGAGGTGGGATG
**Snail 2**	GGGGAGAAGCCTTTTTCTTG	TCCTCATGTTTGTGCAGGAG
**Twist 1**	GGCTCAGCTACGCCTTCTC	CCTTCTCTGGAAACAATGACATCT
**Twist 2**	CCTCTGACAAGCTGAGCAAG	GCAGGACCTGGTAGAGGAAG
**Zeb 1**	AACTGCTGGGAGGATGACA	TCCTGCTTCATCTGCCTGA
**Zeb 2**	CGATCCAGACCGCAATTAAC	TGCTGACTGCATGACCATC
**MRCKα**	TGAATACGCCTACCGATGCT	GTGAGTCTTGCGCTTAGGTG
**MRCKβ**	CCGGAAGATATGGCGAGGTTC	CCATTCACGTCCAAAAGGACAT
**GAPDH**	TGCCTCCTGCACCACCAACT	CCCGTTCAGCTCAGGGATGA
**HPRT1**	TGACCTTGATTTATTTTGCATACC	CGAGCAAGACGTTCAGTCCT
**U1snRNA**	GGGAGATACCATGATCACGAAGGT	ATGCAGTCGAGTTTCCCACA

Supplementary Table 5: List of siRNA oligonucleotides used in this study

Oligo name	*Sequence forward (5'-3')*	*Sequence reverse (5'-3')*
**MMP14-1**	CACAAGGACUUUGCCUCUGAA	UUCAGAGGCAAAGUCCUUGUG
**MM14-2**	AACAGGCAAAGCUGAUGCAGA	UCUGCAUCAGCUUUGCCUGUU
**Snail1-1**	GGACUUUGAUGAAGACCAU	AUGGUCUUCAUCAAAGUCC
**Snail1-2**	AAUCGGAAGCCUAACUACA	UGUAGUUAGGCUUCCGAUU
**Zeb1-1**	CAGUGAAAGAGAAGGGAAU	AUUCCCUUCUCUUUCACUG
**Zeb1-2**	AACUGAACCUGUGGAUUAU	AUAAUCCACAGGUUCAGUU
**Twist1-1**	GGACAAGCUGAGCAAGAUU	AAUCUUGCUCAGCUUGUCC
**Twist1-2**	GGUACAUCGACUUCCUCUA	UAGAGGAAGUCGAUGUACC
**Cdc42-1**	CCGGUGGAGAAGCUGAGGUCAUCAU	AUGAUGACCUCAGCUUCUCCACCGG
**Cdc42-2**	AAAGACUCCUUUCUUGCUUGU	ACAAGCAAGAAAGGAGUCUUU
**RhoA-1**	CGACAGCCCUGAUAGUUUA	UAAACUAUCAGGGCUGUCG
**RhoA-2**	GACCAAAGAUGGAGUGAGA	UCUCACUCCAUCUUUGGUC
**RhoC-1**	AUAAGAAGGACCUGAGGCA	UGCCUCAGGUCCUUCUUAU
**RhoC-2**	GGAUCAGUGCCUUUGGCUA	UAGCCAAAGGCACUGAUCC
**Rac1-1**	UAAGGAGAUUGGUGCUGUA	UACAGCACCAAUCUCCUUA
**Rac1-2**	UAAAGACACGAUCGAGAAA	UUUCUCGAUCGUGUCUUUA
**MRCKα**	CUUAUACCUGGUUAUGGAUUAUUAU	AUAAUAAUCCAUAACCAGGUAUAAG
**MRCKβ**	UAAAUCACCACCCACAUAGUAAUCC	GGAUUACUAUGUGGGUGGUGAUUUA
**ROCK1-2**	GCCAAUGACUUACUUAGGA	UCCUAAGUAAGUCAUUGGC
**ROCK1-1**	CCAGGAAGGUAUAUGCUAU	AUAGCAUAUACCUUCCUGG
**ROCK2-1**	UAGAAUAUGUGGCCUAGAA	UUCUAGGCCACAUAUUCUA
**ROCK2-2**	CAAACUUGGUAAAGAAUUG	CAAUUCUUUACCAAGUUUG
**NWASP-1**	CAGCAGAUCGGAACUGUAU	UACCAAGAAACUCCCGUAC
**NWASP-2**	UAGAGAGGGUGCUCAGCUA	UAGCUGAGCACCCUCUCUA
**Arp3-1**	GGACAGAGUCACAGUAGUC	GACUACUGUGACUCUGUCC
**Arp3-2**	GUACGGGAGUUUCUUGGUA	UACCAAGAAACUCCCGUAC
