# Supplementary material for: EFA6B regulates a stop signal for collective invasion in breast cancer
Source: Nat Commun. 2021 Apr 13;12:2198. doi: 10.1038/s41467-021-22522-4 (PMC8044243; doi:10.1038/s41467-021-22522-4)
Supplement: Supplementary file 3 — Description of Additional Supplementary Files [file 41467_2021_22522_MOESM3_ESM.pdf]

Description of Additional Supplementary Files

File Name: Supplementary Data 1
Description: List of 296 genes differentially expressed between the *PSD4* KO MCF10A cells (N=5, including KO55 (N=3) and KO2.9 cells (N=2)) and the *PSD4* WT MCF10A cells (N=3)

File Name: Supplementary Data 2
Description: Gene ontologies of the 296 genes differentially expressed between the *PSD4* KO MCF10A cells (N=5, including KO55 (N=3) and KO2.9 cells (N=2)) and the *PSD4* WT MCF10A cells (N=3)

File Name: Supplementary Movie 1
Description: 64h time-lapse imaging at 20X magnification. Left panels: top MCF10A WT (2h time points), bottom MCF10A SgCtl (4h time points). Middle panels: EFA6B KO2 (top panel, 4h time points; bottom panel, 2h time points), right panels EKA6B KO55 (2h time points). n=62.

File Name: Supplementary Movie 2
Description: 64h or 120h time-lapse imaging at 4X magnification of the same two movies of EFA6B KO2 presented at 20X magnification. n=23.
