# Supplementary material for: EFA6B regulates a stop signal for collective invasion in breast cancer
Source: Nat Commun. 2021 Apr 13;12:2198. doi: 10.1038/s41467-021-22522-4 (PMC8044243; doi:10.1038/s41467-021-22522-4)
Supplement: Supplementary file 8 — Reporting Summary [file 41467_2021_22522_MOESM8_ESM.pdf]

nature research

Corresponding author(s): Luton Frédéric

Last updated by author(s): YYYY-MM-DD

Reporting Summary

Nature Research wishes to improve the reproducibility of the work that we publish. This form provides structure for consistency and transparency in reporting. For further information on Nature Research policies, see our Editorial Policies and the Editorial Policy Checklist.

Statistics

For all statistical analyses, confirm that the following items are present in the figure legend, table legend, main text, or Methods section.

n/a	Confirmed	
☐	☒	The exact sample size (*n*) for each experimental group/condition, given as a discrete number and unit of measurement
☐	☒	A statement on whether measurements were taken from distinct samples or whether the same sample was measured repeatedly
☐	☒	The statistical test(s) used AND whether they are one- or two-sided<br>*Only common tests should be described solely by name; describe more complex techniques in the Methods section.*
☐	☒	A description of all covariates tested
☐	☒	A description of any assumptions or corrections, such as tests of normality and adjustment for multiple comparisons
☐	☒	A full description of the statistical parameters including central tendency (e.g. means) or other basic estimates (e.g. regression coefficient) AND variation (e.g. standard deviation) or associated estimates of uncertainty (e.g. confidence intervals)
☐	☒	For null hypothesis testing, the test statistic (e.g. *F*, *t*, *r*) with confidence intervals, effect sizes, degrees of freedom and *P* value noted<br>*Give P values as exact values whenever suitable.*
☐	☒	For Bayesian analysis, information on the choice of priors and Markov chain Monte Carlo settings
☒	☐	For hierarchical and complex designs, identification of the appropriate level for tests and full reporting of outcomes
☒	☐	Estimates of effect sizes (e.g. Cohen's *d*, Pearson's *r*), indicating how they were calculated

Our web collection on statistics for biologists contains articles on many of the points above.

Software and code

Policy information about availability of computer code

Data collection

Patients and transcriptomic data: R package v3.5.2 and v3.5.3, Limma R package v3.38.3, survival R package v3.1.12, qvalue R package v2.14.1, oligo R package 1.46.0, affy v1.60, MSigDB v5.2.
Flow cytometry: BD LSRII FortessaTM cell analyser.
IF images were acquired with TCS SP5 confocal (Leica).
Immunoblot images were acquired with a Fusion Fx Vilbert Lourmat and its software FusionCapt Advance FX7
Time-lapse images were acquir ved with the Cytation5 Biotek and ts software Gen5 v2.09.2
RT-qPCR were run with the LightCycler480.
Alamar bue-based assay were acquired using the microplate reader Infinite M1000 Pro, TECAN
IHC images acquisition were acquired with a Scanner Hamamatsu Nanozoomer 2.0 HT and its software CaloPix Research v4.1.0.6

Data analysis

Affymetrix transcriptomic data were analyzed in R software (version 3.5.3, http://www.cran.r-project.org/). Data were normalized by Robust Multi-Array (RMA) with the oligo R package (version 1.46.0). Prior to analysis, expression data were filtered to remove probes with low and poorly measured expression and standard deviation inferior to 0.25 log2 units across samples. Supervised analyis was done using a moderated t-test with empirical Bayes statistic included in the limma R package (version 3.38.3). For correction of the multiple-testing hypothesis, False Discovery Rate (FDR) was assessed using qvalue R package (version 2.14.1) (Storey et al., Annals of Statistics, 2003). Enrichment analysis was done with Gene Set Enrichment Analysis (GSEA, version 4.0.3) (http://www.broadinstitute.org/gsea/).
Statistics related to transcriptomic data were done with the stats R package (version 3.5.2) and the survival R package (version 3.1-12) for survival analysis. All other statistical analyses have been performed with GraphPad Prism software (version 8 and 9).
Time-lapse imaging films were prepared with FilmoraPro v.2.3.10723.54848
Fow cytometry results were processed using Kaluza (v2.1) and FlowJo (v10) softwares.
Confocal images were processed and analyzed with ImageJ/FIJI and AdobePhotoshop CS2 v9.0.
Immunoblot images were quantified with AIDA v4.27.039
RT-qPCR data were analyzed wth LightCycler software v1.5.

For manuscripts utilizing custom algorithms or software that are central to the research but not yet described in published literature, software must be made available to editors and reviewers. We strongly encourage code deposition in a community repository (e.g. GitHub). See the Nature Research guidelines for submitting code & software for further information.

Data

Policy information about availability of data

All manuscripts must include a data availability statement. This statement should provide the following information, where applicable:

- Accession codes, unique identifiers, or web links for publicly available datasets
- A list of figures that have associated raw data
- A description of any restrictions on data availability

Generated microarray data were deposited on Array-Express repository (E-MTAB-10063, https://www.ebi.ac.uk/arrayexpress/). For public data sets of invasive breast cancer, all data analyzed were downloaded from the Gene Expression Omnibus (GEO, https://www.ncbi.nlm.nih.gov/geo/), Genomic Data Commons (GDC, https://portal.gdc.cancer.gov/) and European Genome-phenome Archive (EGA, https://ega-archive.org/) databases as detailed in Supplementary Table 3. DCIS public data sets were downloaded from GEO (GSE41228 and GSE33692 for Lee's and Knudsen's data sets respectively. , A detailed description of the pre-analytic transcriptomic data processing for public data sets is linked in our manuscript (Zangari et al., Cancer Res 2014). In details, the first step was the normalization of each set separately. It was done in R using Bioconductor and associated packages i.e. limma (version 3.38.3), affy (version 1.60); we used quantile normalization for the available processed data from non-Affymetrix-based sets (Agilent, SweGene, and Illumina), and Robust Multichip Average (RMA) with the non-parametric quantile algorithm for the raw data from the Affymetrix-based sets. In the second step, we mapped the hybridization probes across the different technological platforms represented as previously reported.(Bertucci et al, Mol Cancer 2014) When multiple probes mapped to the same GeneID, we retained the most variant probe in a particular dataset. We log2-transformed the available TCGA RNA-Seq data that were already normalized. Enrichment analysis with GSEA was done using GO and KEGG terms included in the Molecular Signature Database (MSigDB version 5.2, http://www.gsea-msigdb.org/gsea/msigdb/index.jsp) and the human matrisome database (version August 2014; Hynes Lab; Naba A, Ding H, Whittaker CA, Hynes RO. http://matrisomeproject.mit.edu).

Field-specific reporting

Please select the one below that is the best fit for your research. If you are not sure, read the appropriate sections before making your selection.

☒ Life sciences ☐ Behavioural & social sciences ☐ Ecological, evolutionary & environmental sciences

For a reference copy of the document with all sections, see nature.com/documents/nr-reporting-summary-flat.pdf

Life sciences study design

All studies must disclose on these points even when the disclosure is negative.

Sample size

Regarding, the transcriptomic analyses, the sample size was determined by availability of biological materials. Quantification of all the cell functional assays (invasion, gelatin degradation, invadopodia formation) was performed at least in 3 independent experiments with a minimal size population of each group of 30 samples (cells or cell aggregates) per experiment. At n=30, a normal distribution of the population is assumed (verified by the sample variance (SD)) and justifies the use of the student t-test

Data exclusions

No data were excluded.

Replication

For transcriptomic analyses, the reproducibility of our results was assessed on external data sets. All cell experiments were repeated at least 3 times with satisfactory reproducibility as indicated by the SEM and p-values of the Student t-test. The new in vivo experiments were performed once at week 2,3,6,7 and twice at week 4,5. All attempts at replication were successful.

Randomization

Randomization is not relevant to most biochemical and cell experiments such as immuno-blot, pull-down, RT-qPCR or FACS as large number of cells are analyzed in a random manner by the nature of the experimental procedure. For all microscopy studies, fields were analyzed through a consistent pattern applied to each sample, and all cells or cell agregates within each field were analyzed. For IF co-localization quantification a Coste randomization test was run as described in the Method section.
Human research participants: Patients population characteristics: Our study used public data from published studies on breast cancer in which population characteristics are detailed and could be found using accession codes provided in Supplementary Table 3. All cases were invasive breast carcinomas profiled using DNA microarrays or RNA-sequencing with expression and clinicopathological data available. All samples were pre-treatment samples (operative specimen or diagnostic biopsy before neo-adjuvant chemotherapy). The characteristics of patients analyzed in the study are available in the supplementary table 4. Patients were characterized upon patients' age at diagnosis (≤50 years, >50), pathological grade (1, 2, 3), pathological type (lobular, ductal, other), pathological axillary lymph nod status (pN: negative, positive), pathological tumor size (pT1, pT2, pT3), molecular subtype (HR+/HER2-, HER2+, TN), and disease-free survival (DFS) with follow-up.
Recruitment: Our study is based upon publicly available transcriptomic data of invasive primary breast cancer enrolled in 5 retrospective studies published over a period between 2010 and 2012. The data collection was done in our laboratory in real time after each publication. The biases are those of every retrospective study. But importantly the present series is completely independent from the previous large series we published in Cancer Research (Zangari et al. 2014).
Xenograft experiments were conducted only in female as we are studying breast cancer. No special randomization protocol was used: mice were used as littermates and randomly assigned to given subgroups.

Blinding	Blinding to the data patients and tumor tissue was not necessary because the analyses were automated. Investigators were blinded to group allocation during data collection for most invasion and invadopodia formation assays. All other data analyses were based on objectively measurable data (fluorescence intensity, gene expression level, object size, density,...). For these particular experiments, care was taken to ensure all samples were uniformly processed and analyzed for a consistency of the results. Also, all key experiments were repeated at least once by two independent investigators. Since these datasets are primarily quantitative and not subjective, blinding was not necessary.

Reporting for specific materials, systems and methods

We require information from authors about some types of materials, experimental systems and methods used in many studies. Here, indicate whether each material, system or method listed is relevant to your study. If you are not sure if a list item applies to your research, read the appropriate section before selecting a response.

Materials & experimental systems

n/a	Involved in the study	
☐	☒	Antibodies
☐	☒	Eukaryotic cell lines
☒	☐	Palaeontology and archaeology
☐	☒	Animals and other organisms
☐	☒	Human research participants
☒	☐	Clinical data
☒	☐	Dual use research of concern

Methods

n/a	Involved in the study	
☒	☐	ChIP-seq
☐	☒	Flow cytometry
☒	☐	MRI-based neuroimaging

Antibodies

Antibodies used

The supplementary table S5 list all the antibodies used, with reference, source, application and correspondingi dilution/concentration
Integrin-β1 Santa Cruz, sc-53711
Integrin-β1 Santa Cruz, sc-13590
Integrin-β4 BD Biosciences, 611232
Integrin-β4 BD Biosciences, 555719
Integrin-β4 Millipore, MAB2059
Integrin-α2 Santa Cruz, sc-53502
Integrin-α2 Millipore, MAB1950
Integrin-α3 Millipore, MAB2057
Integrin-α6 BD Biosciences, 555734
EpCAM BD Biosciences, 563181
CD49f BD Biosciences, 562582
CD24 BD Biosciences, 561644
CD44 BD Biosciences, 560568
N-cadherin BD Biosciences, 610921
E-cadherin Thermo Scientific, 33-4000
E-cadherin BD Biosciences, 610182
Vimentin Sigma, V6389
Claudin 1 ZYMED Laboratories, 51-9000
Claudin 3 Millipore, 2819163
Occludin Thermo Scientific, 40-4700
ARF1 Novus Biologicals, NB100-55421
ARF5 Abnova, H00000381-M01
ARF6 Gift from Dr. Bourgoin
EFA6A Gift from Dr. Sakagami
EFA6B Sigma, HPA034722
EFA6D Gift from Dr. Sakagami
MMP-14 Millipore, MAB3328
Cortactin Millipore, 05-180
N-WASP Cell Signaling, 4848
ARP3 BD Biosciences, 612234
pMLC Cell Signaling, 3671 & 3675
MLC Sigma, M4401

CDC42 BD Biosciences, 610928
RAC1 BD Biosciences, 610650
RHOA Santa Cruz, sc-418
RHOC Cell Signaling, 3430
ROCK 1 Santa Cruz, sc-17794
ROCK 2 Santa Cruz, sc-398519
P63 Diagomics, BSB3606
Actin Sigma, A4700
Actinin Sigma, A5044
GST GE Healthcare, 27-4577-01
Hsp60 Sigma, SAB4501464
p85 Millipore, ABS1856
GAPDH Sigma, G9545

Validation

The information can be found in the method section. For all the antibodies, additional information including specificity, species crossreactivity, with links to key publications can be found on the distributor's websites. The antibodies are validated for the indicated use by the manufacturer and details available on their websites.
In brief, here are the manufacturer's recommandation, references and validation statement conducted in this study.
Integrin-β1 Santa Cruz, sc-53711 Anti-Integrin beta 1 Antibody (TS2/16) is recommended for detection of Integrin β1 of mouse, rat and human origin by WB, IP, IF and FCM; also reactive with additional species, including and bovine and canine
Integrin-β1 Santa Cruz, sc-13590 Integrin beta 1 Antibody (P5D2) is recommended for detection of Integrin β1 of human origin by WB, IP, IF and FCM
Integrin-β4 BD Biosciences, 611232 Western blot (Routinely Tested)
Immunofluorescence (Tested During Development)
Integrin-β4 BD Biosciences, 555719 Flow cytometry (Routinely Tested)
Integrin-β4 Millipore, MAB2059 Reactivity: human. Key applications: FC, IP, IHC, FUNC
Integrin-α2 Santa Cruz, sc-53502 recommended for detection of Integrin α2 of human origin by WB, IP and IF
Integrin-α2 Millipore, MAB1950 Reactivity: human. Key applications: ICC, IHC, FUNC
Integrin-α3 Millipore, MAB2057 Reactivity: human. Key applications: FC, IP, IHC, FUNC
Integrin-α6 BD Biosciences, 555734 Flow cytometry (Routinely Tested)
Immunohistochemistry-frozen, Immunoprecipitation (Reported)
EpCAM BD Biosciences, 563181 Flow cytometry (Routinely Tested)
CD49f BD Biosciences, 562582 Flow cytometry (Routinely Tested)
CD24 BD Biosciences, 561644 Flow cytometry (Routinely Tested)
CD44 BD Biosciences, 560568 Flow cytometry (Routinely Tested)
N-cadherin BD Biosciences, 610921 Western blot (Routinely Tested)
Immunofluorescence (Tested During Development)
Immunoprecipitation (Reported)
E-cadherin Thermo Scientific, 33-4000 Species Human, Published Species Dog, Avian, Rat, Pig, Hamster, Human, Mouse. Applications: IHC, WB, IP, ChIP, ICC, IF.
E-cadherin BD Biosciences, 610182 Western blot (Routinely Tested)
Immunoprecipitation, Immunofluorescence, Immunohistochemistry (Tested During Development)
Vimentin Sigma, V6389 Recognizes human, monkey,pig, rat, and chicken5 vimentin. The antibody may be used in immunoblotting, immuno-cytochemistry, immunohistochemistry,and flow cytometry
Claudin 1 ZYMED Laboratories, 51-9000 Species: Chicken, Human, Rat
Published species
Bovine, C. elegans, Chicken, Dog, Hamster, Human, Mouse, Non-human primate, Pig, Rat, Tag, Virus. Applications: WB, IHC, ICC, IF, ELISA, IP.
Claudin 3 Millipore, 2819163 Quality Control Testing
Evaluated by Western Blotting in HUVEC cell lysate.
Occludin Thermo Scientific, 40-4700 Species: Dog, Human, Mouse, Rat. Published species: Pig, Rat. Applications: WB, IHC, ICC, IF, IP
ARF1 Novus Biologicals, NB100-55421 Reactivity: Hu, Mu, Rt, Ca. Applications: WB, PEP-ELISA
ARF5 Abnova, H00000381-M01 Reactivity:Human, Mouse, Rat. Applications: ELISA, IF, WB
ARF6 Gift from Dr. Bourgoin Application: WB, validated in PMID: 9150938
EFA6A Gift from Dr. Sakagami Application WB validated in PMID: 17298598
EFA6B Sigma, HPA034722 Reactivity: human. Applications:ICC, WB (validated in this study by KO EFA6B)
EFA6D Gift from Dr. Sakagami Application WB validated in PMID: 16707115
MMP-14 Millipore, MAB3328 Reactivity: human, mouse. Key applications: ELISA, IP, WB validated in this study by siRNA knock-down
Cortactin Millipore, 05-180 Reactivity: Av, B, H, Ht, M, R . Key applications: ICC, IHC, IP, WB
N-WASP Cell Signaling, 4848 Reactivity: Hu, Mo, Rat, Application: IP, WB: validated in this study by siRNA knock-down
ARP3 BD Biosciences, 612234 Western blot (Routinely Tested)
Immunofluorescence (Tested During Development). WB: validated in this study by siRNA knock-down
pMLC Cell Signaling, 3671 & 3675 Reactivity: Hu, Mo, Rat. Application: WB, IF, ICC.
MLC Sigma, M4401 Reactivity: chicken, pig, rabbit, bovine, human. Applications: WB, IF and ICC
CDC42 BD Biosciences, 610928 Western blot (Routinely Tested)
Immunofluorescence (Not Recommended). WB: validated in this study by siRNA knock-down
RAC1 BD Biosciences, 610650 Western blot (Routinely Tested)
Immunofluorescence, Immunohistochemistry (Tested During Development)
Immunoprecipitation (Not Recommended). WB: validated in this study by siRNA knock-down
RHOA Santa Cruz, sc-418 Anti-Rho A Antibody (26C4) is recommended for detection of Rho A of mouse, rat and human origin by WB, IP, IF, IHC(P) and FCM; also reactive with additional species, including and equine, bovine, porcine and canine. WB: validated in this study by siRNA knock-down
RHOC Cell Signaling, 3430 Reactivity: Hu, Mo, Mk. Application WB: validated in this study by siRNA knock-down
ROCK 1 Santa Cruz, sc-17794 Anti-Rock-1 Antibody (G-6) is recommended for detection of Rock-1 of mouse, rat and human origin by WB, IP, IF, IHC(P) and ELISA WB: validated in this study by siRNA knock-down

ROCK 2 Santa Cruz, sc-398519 Anti-Rock-2 Antibody (D-11) is recommended for detection of Rock-2 of mouse, rat and human origin by WB, IP, IF and ELISA WB: validated in this study by siRNA knock-down
P63 Diagomics, BSB3606 Reactivity: Hu, Mo, rat. Applications: IHC
Actin Sigma, A4700 Reactivity: human, carp, Xenopus, canine, sheep, pig, mouse, rabbit, rat, hamster, chicken, snail, bovine, viper, guinea pig, goat. Applications: ICC, ELISA, IF, WB.
Actinin Sigma, A5044 Reactivity: human, chicken, bovine, mouse. Applications: WB, IF, ICC. WB: validated by siRNA knock-down in PMID: 29246944
GST GE Healthcare, 27-4577-01 Reactivity: schistosomal GST. Applications: WB, dot blot.
Hsp60 Sigma, SAB4501464 Reactivity: human, rat, mouse. Applications: ELISA, IF, ICC, WB
p85 Millipore, ABS1856 Reactivity: human . Key applications: WB, IP
GAPDH Sigma, G9545 Reactivity: mouse, human, rat. Applications: IP, IF, WB

Eukaryotic cell lines

Policy information about cell lines

Cell line source(s)

MCF10A and HEK-293T from ATCC, HMLE from Dr. R.A. Weinberg (Whitehead Institute for Biomedical Research, Cambridge, MA, USA), MCF10 DCIS.com from Asterand were obtained from Dr. P. Chavrier (Institut Curie, Paris, France).

Authentication

MCF10A and HEK-293T were authenticated by short tandem repeat profiling by the vendor. MCF10A cell line was controlled upon pangenome expression profiles by hierarchical clustering including as reference 33 cells lines including MCF10A from previoulsy data published (Charafe-Jauffret et al., CR 2009). All MCF10A experiments of this study were strongly clustered with the MCF10A reference. The HMLE cell population was regularly tested by FACS using the four markers CD44, CD24, CD9f and EpCAM to insure the presence of all three mammary epithelial populations and the absence of contamination by other cell lines cultivated in our laboratory. DCIS.com cells were not authenticated. All cells were expanded upon arrival to store large stocks of original samples in liquid nitrogen. Each cell lines were passaged only 10 times at which point a new vial was thawed to avoid any derivation or contamination.

Mycoplasma contamination

We perform regularly a PCR assay to look for the presence of Mycoplasma in our cultures and never found any contamination. In addition, DAPI staining never revealed any sign of contamination of our cell lines

Commonly misidentified lines
(See ICLAC register)

No commonly misindentified cell lines were used in this study

Animals and other organisms

Policy information about studies involving animals; ARRIVE guidelines recommended for reporting animal research

Laboratory animals

Nude SCID mice female 8 week old

Wild animals

No wild animals were used in the study.

Field-collected samples

No field collected samples were used in the study.

Ethics oversight

All experiments were done in agreement with the French Guidelines for animal handling and approved by the local ethics committee: Comité d'Ethique en Expérimentation Animal(CIEPAL, Agreement n° 2016091517253478).

Note that full information on the approval of the study protocol must also be provided in the manuscript.

Human research participants

Policy information about studies involving human research participants

Population characteristics

Our study used public data from published studies on breast cancer in which population characteristics are detailed and could be found using accession codes provided in Supplementary Table 3. All cases were invasive breast carcinomas profiled using DNA microarrays or RNA-sequencing with expression and clinicopathological data available. All samples were pre-treatment samples (operative specimen or diagnostic biopsy before neo-adjuvant chemotherapy). The characteristics of patients analyzed in the study are available in the supplementary table 4. Patients were characterized upon patients' age at diagnosis (≤50 years, >50), pathological grade (1, 2, 3), pathological type (lobular, ductal, other), pathological axillary lymph node status (pN: negative, positive), pathological tumor size (pT1, pT2, pT3), molecular subtype (HR+/HER2-, HER2+, TN), and disease-free survival (DFS) with follow-up.

Recruitment

Our study is based upon publicly available transcriptomic data of invasive primary breast cancer enrolled in 5 retrospective studies published over a period between 2010 and 2012. The data collection was done in our laboratory in real time after each publication. The biases are those of every retrospective study. But importantly the present series is completely independent from the previous large series we published in Cancer Research (Zangari et al. 2014).

Ethics oversight

Public data come from published studies in which the patients consent to participate, ethics and institutional review board were already obtained by the authors. Our study was approved by our institutional review board (Comité d'Orientation Stratégique, COS).

Note that full information on the approval of the study protocol must also be provided in the manuscript.

Flow Cytometry

Plots

Confirm that:

☒ The axis labels state the marker and fluorochrome used (e.g. CD4-FITC).

☒ The axis scales are clearly visible. Include numbers along axes only for bottom left plot of group (a 'group' is an analysis of identical markers).

☒ All plots are contour plots with outliers or pseudocolor plots.

☒ A numerical value for number of cells or percentage (with statistics) is provided.

Methodology

Sample preparation	Only cell lines grown in vitro were studied and prepared as described in the Methods section and as follows: cells were detached using Accutase (Stemcell technologies, NC, USA) and washed 3 times in PFE (PBS, 2mM EDTA, 2% Foetal Bovine Serum). The cells were incubated at 4°C for 30 min in the presence of the indicated antibodies diluted in PFE. After washes, cells resuspended in cold PFE were examined by BD LSRII FortessaTM cell analyser. The ALDEFLUOR kit (Stemcell Technologies) was used to quantify the ALDH enzymatic activity. Results were processed using Kaluza v2.1 or FlowJo v10 softwares.
Instrument	For sorting Fig2a Becton Dickinson FACS Aria Ill and for FACS analysis Fig4a Becton Dickinson LSRII Fortessa
Software	Kaluza v2.l (Fig2a, SuppFig3c) and FlowJo v10 (Fig4a)
Cell population abundance	Since using cell lines we were not limited by the number of cells
Gating strategy	For sorting (Fig2a) and FACS analysis (SuppFig3c): first FSC-A/SSC-A to select living cells, then quadrant positioning by FMO followed by FSC-A/FSC-H for doublet exclusion of each separated populations. We used antibodies directly coupled to a fluorochrome: EpCAM-BV510 and CD49f-Pacific Blue.<br>For FACS analysis Fig4a, first FSC-A/SCC-A, then FSC-A/FSC-H, then quadrant positioning based on signal of the corresponding fluorescent secondary antibody alone.we used secondary fluorescent antibodies coupled to Alexa488 for all primary anti-integrin antibodies.

☒ Tick this box to confirm that a figure exemplifying the gating strategy is provided in the Supplementary Information.
